# Multifunctional Nanomaterials: Synthesis, Properties and Applications

**DOI:** 10.3390/ijms222112073

**Published:** 2021-11-08

**Authors:** Raghvendra Singh Yadav

**Affiliations:** Centre of Polymer Systems, Tomas Bata University in Zlin, Trida Tomase Bati 5678, 76001 Zlin, Czech Republic; yadav@utb.cz; Tel.: +420-576031725

In this Special Issue “*Multifunctional Nanomaterials: Synthesis, Properties and Applications*”, we published three review papers and nine original research articles.

In the first article (review) [1], Si Amar Dahoumane et al. describe the recent advances in the emerging approaches applied for the biosynthesis of selenium and tellurium nanoparticles using different microorganisms, such as bacteria and fungi, and plant extracts. The green synthesis approach provides prospects to develop safer nanomaterials and also promote a better understanding of safety, health, and environment concerns. The second (review) article by Joan Estelrich et al. [2] reviews the enzyme-like properties of Prussian blue nanoparticles (PBNPs) and their applications in this field for biomedicine development. In the third (review) article [3], Marika Musielak et al. presented the most recent progress in the utilization of advanced nanostructures such as liposomes combined with metallic nanoparticles and other active substances such as drugs or contrast agents.

In the fourth article (research), Rafael Bosch et al. [4] reported the effective elimination and biodegradation of polycyclic aromatic hydrocarbons from seawater through the development of magnetic microfibers. Joanna Kolmas et al. [5] reported the Mg, Si-Co-substituted hydroxyapatite/alginate composite beads loaded with raloxifene for bone tissue regeneration application. The sixth article (research) by Amjad Ali et al. [6] discussed the Cu and Cu-SWCNT nanoparticle suspension in pulsatile casson fluid flow via a Darcy-Forchheimer porous channel with compliant walls. In the seventh article (research) [7], Yuan-Chang Liang et al. reported the development of a brush-like Fe_2_O_3_–ZnO nanostructures towards NO_2_ gas sensing based on the control of appropriate material synergistic effects. Further, Zhikun Peng et al. [8] reported the influence of ZnSO_4_, MnSO_4_ and FeSO_4_ on the partial hydrogenation of benzene over nano Ru-Based catalysts. The ninth published research article by Xiaoyu Yu et al. [9], discussed the development of an optimized synthesis approach for ZnO quantum dots (QDs) modified with polyvinylpyrrolidone (PVP40) which can potentially be applied in the fields of photoluminescence and photodynamic tumor suppression. In the tenth research article of this Special Issue, Xiang Zhao et al. [10] report a novel, versatile and controllable approach for efficient and safe nanopesticides by the anodic aluminum oxide templates-assisted method.

Furthermore, in the eleventh published research article, Yuan Tan et al. [11] reported that Zn promoted Mg-Al mixed oxides-supported gold nanoclusters for the direct oxidative esterification of aldehyde to ester. In the last, i.e., twelfth, published research article, Florian Grüner et al. [12] discuss a numerical study on the feasibility of monitoring tumor response by tracking nanoparticle-labelled T cells by utilizing the X-ray fluorescence imaging technique.

## Data Availability

Not applicable.
